# Procedure for Detecting Outliers in a Circular Regression Model

**DOI:** 10.1371/journal.pone.0153074

**Published:** 2016-04-11

**Authors:** Adzhar Rambli, Ali H. M. Abuzaid, Ibrahim Bin Mohamed, Abdul Ghapor Hussin

**Affiliations:** 1 Institute of Mathematical Sciences, University of Malaya, Kuala Lumpur, Malaysia; 2 Department of Mathematics, Faculty of Science, Al-Azhar University-Gaza, Palestine; 3 Centre for Defence Foundation Studies, National Defence University of Malaysia, Kuala Lumpur, Malaysia; National Institute of Environmental and Health Sciences, UNITED STATES

## Abstract

A number of circular regression models have been proposed in the literature. In recent years, there is a strong interest shown on the subject of outlier detection in circular regression. An outlier detection procedure can be developed by defining a new statistic in terms of the circular residuals. In this paper, we propose a new measure which transforms the circular residuals into linear measures using a trigonometric function. We then employ the row deletion approach to identify observations that affect the measure the most, a candidate of outlier. The corresponding cut-off points and the performance of the detection procedure when applied on Down and Mardia’s model are studied via simulations. For illustration, we apply the procedure on circadian data.

## Introduction

The occurrence of outliers in a data set has been widely discussed in the literature. Their occurrence may be due to error, or part of the phenomena under study. Either way, it is important to identify outliers so that further investigation can be conducted. In linear regression, extensive study on the problem of outliers and leverage points can be found in the literature (e.g. [1, 2, 3]). Many statistical software packages provide different tools to identify outliers in linear regression models. However, such studies are rarely found for circular regression models where the dependent and independent variables are of circular form.

Circular variables are commonly found in many scientific fields such as meteorology. The variable takes the values in the range [0,2*π*) radian. The existence of outliers in circular data may affect the estimation of the parameters and weaken the accuracy of forecasting. Thus, it is of interest to develop suitable methods of identifying outliers in circular problem. We focus on developing such method for circular regression model.

The regression of a circular dependent variable on a set of linear variables was first discussed by Gould [4]. The model follows closely the linear regression form and an iterative method was used to estimate the parameters by maximizing the likelihood function, with further improvement made by Fisher and Lee [5] and Johnson and Wehrly [6]. On the other hand, the first attempt to fit a circular regression models of two circular variables *u* and *v* was made by Laycock [7] using the complex linear regression, where the model can be expressed as a conventional linear model with complex entries. Rivest [8] proposed another regression model with specific application in predicting the direction of earthquake displacement. On the other hand, Jammalamadaka and Sarma [9] expressed a circular-circular model in terms of Fourier series expansions while Hussin et. al [10] assumed the two circular variables are related in linear form. In this paper, we consider the circular regression model proposed by Downs and Mardia [11], and would refer the model as "DM circular regression model" for the rest of the paper.

Although the first discussion of circular regression goes back to Gould [4], there are few known published work found on the identification of outliers in circular regression. Abuzaid et al. [12] and Ibrahim et al. [13] explored the problem on two types of circular regression models by observing the effect of removing one observation on the covariance matrix. Further, Abuzaid et al. [14] proposed a residual measure using a cosine function to detect outliers in a linear circular regression model, where the relationship between the dependent and independent variables is strictly linear (see [10]). In this paper, we propose a new summary measure for the purpose of detecting outliers in terms of a simple measure of circular distance in DM circular regression model. Due to the compact close range of circular variables, it is expected that the effect of masking problem is minimal.

With that view in mind, this paper is organized as follows: Firstly, we review the theory of DM circular regression models. Secondly, the proposed statistic to be used in identifying influential observations in DM circular regression models is presented. Thirdly, we conduct simulation studies to investigate the sampling behavior of the statistic and the performance of the procedure of detecting influential observation. Finally, we then apply the procedure on the circadian data as given in Down and Mardia [11].

### DM Circular Regression Model

Assume that (*u*,*v*) are a pair of independent and dependent random angles with angular location parameters *α* and *β* respectively, and *ω* is a slope parameter in the closed interval [–1, 1]. Down and Mardia [11] proposed the DM circular regression model given by

$$tan\frac{1}{2}(v-\beta )=\omega tan\frac{1}{2}(u-\alpha ).$$ (1)

The model ensures a one-to-one relationship between *u* and *v*, *ω* ≠ 0. The relationship can be described by a continuous closed curve winding around a toroidal surface. The model has a unique solution given by

$$v=\beta +2{tan}^{-1}\{\omega tan\frac{1}{2}(u-\alpha )\}.$$ (2)

Suppose that *v* in Eq (2) is replaced by *μ*, the mean direction for *v* given*u*. The resulting DM circular regression model is given by

$$\text{tan}\frac{1}{2}\left(\mu -\beta \right)=\omega \text{tan}\frac{1}{2}\left(u-\alpha \right)$$ (3)

which has a unique solution

$$\mu =\beta +2{tan}^{-1}\{\omega tan\frac{1}{2}(u-\alpha )\}.$$ (4)

As can be seen, the model has three functionally independent parameters*α*, *β* and *ω*. It can be shown that the log-likelihood function for a random sample of *n* pairs (*u*_*j*_, *v*_*j*_), *j* = 1, 2,…,*n*, is

$$\begin{array}{lll} l\left(\alpha ,\beta ,\omega ;v_{1},\ldots ,v_{n}\right) & = & -n\text{log}I_{0}\left(\kappa \right)\\ & & \;+\kappa \sum_{j}\text{cos}\left(v_{j}-\beta -v\left(u_{j}-\alpha ;\omega \right)\right)+\text{constant } \end{array}$$ (5)

where *κ* is the concentration parameter, $I_{0}\left(\kappa \right)=\sum_{j=0}^{\infty }{\left({\left(\kappa /2\right)}^{j}/j\;!\right)}^{2}$ is the modified Bessel function of the first kind order zero and $v\left(u_{j}-\alpha ;\omega \right)=2{\text{tan}}^{-1}\left\{\omega \text{tan}\frac{1}{2}\left(u_{j}-\alpha \right)\right\}$. We may define explicitly the maximum likelihood estimator $\hat{\rho }$ of the precision parameter *ρ* by

$$\hat{\rho }(\alpha ,\beta ,\omega )=\frac{1}{n}\sum_{j}cos(v_{j}-\beta -v(u_{j}-\alpha ;\omega )).$$ (6)

Hence, the log-likelihood functions of Eq (5) and maximum likelihood estimator $\hat{\rho }$ of Eq (6) are changed accordingly.

We employ an iterative method of obtaining the estimates of (*α*, *β*, *ω*), say $\left(\hat{\alpha },\hat{\beta },\hat{\omega }\right)$, which maximize Eq (5). This can be done by using the MS function available in S-Plus software. The function requires the determination of initial values *α*_0_, *β*_0_ and *ω*_0_. These initial values can be taken to values which give maximum precision parameter $\hat{\rho }$ in Eq (6) for all possible pairs (*α*, *β*, *ω*) in pre-specified sets. In our case, the following sets of parameter values are considered; *α* = [−*π*, *π*], *β* = [−*π*, *π*] and *ω* = [−1, 1]. Then using those initial values, we obtain the estimates iteratively for the three parameters of the model.

### Definition of a New Statistic

Upon fitting the bivariate circular variables (*u*_*j*_, *v*_*j*_), *j* = 1, 2,…,*n*, we obtain the fitted values of *v*_*j*_, say ${\hat{v}}_{j}$. It is then useful to utilize the fitted values in evaluating the goodness-of-fit of the DM circular regression model in terms of circular errors. One useful measure is the circular distance between two circular observations, say *ϕ* and *θ*, as given by Jammalamadaka and SenGupta [15]. It is defined by as *d*_∘_(*ϕ*, *θ*) = *π*−|*π*−|*ϕ*−*θ*||, *d*_∘_∈[0, *π*]. Down and Mardia [11] in Section 2.3 had shown that the angular error where in our case, the difference between *v*_*j*_ and ${\hat{v}}_{j}$ is then given by $d_{j}=\pi -\left|\pi -\left|v_{j}-{\hat{v}}_{j}\right|\right|$ which can also be treated as a circular error of the model follow a von Mises distribution denoted as *VM* with mean direction *μ* = 0 and concentration parameter*κ*. In measuring the overall goodness-of-fit of the model, we may define a summary measure of errors called mean circular error (*MCEs*) as

$$MCEs=\frac{1}{n}\sum_{j=1}^{n}\text{sin }\left(\frac{d_{j}}{2}\right)$$ (7)

where *n* is the sample size and *MCEs*∈[0, 1].

We intend to use a row deletion method to investigate the effect of removing an observation from the data set on the values of *MCEs*. The effect can be measured by looking at the maximum absolute difference between the value of the statistics for full and reduced data sets, denoted by *DMCEs*, such that

$$DMCEs=\underset{j}{\text{max}}\left\{\;\left|MCEs-MCEs_{\left(-j\right)}\right|\;\right\}$$ (8)

where *MCEs* and *MCEs*_(-*j*)_ are the values of Eq (7) for the full data set and when the *j*th observation is removed from the data, respectively. Any observation will be identified as an outlier if the corresponding value of *DMCEs* exceeds a pre-specified cut-off point.

### Sampling Behavior of the *DMCEs* Statistic

We perform a simulation study to investigate the sampling behavior of the *DMCEs* statistic. A set of circular random errors of sizes *n* = 10, 20, 30, 50, 70, 100 and 150 are generated from a *VM* with mean direction *μ* = 0 and various values of concentration parameter *κ* = 5, 10, and 20. We also generate the values of the independent circular random *u* from *VM*(*π*/2, 3) of size *n*. Observed values of the response variable *v* are then calculated based on the DM circular regression model with fixed values of *α* = 1.5, *β* = 1.5, and *ω* = 0.5. Upon fitting the simulated data, we obtain the fitted values $\hat{v}$ of the DM circular regression model. Then, we compute the values of *MCEs* and *MCEs*_(-*j*)_ for *j* = 1, 2,…, *n*. Hence, the values of the *DMCEs* statistic for every observation are obtained. For each case, the process is carried out 2000 times and the 1%, 5% and 10% upper percentiles of the statistic are calculated as tabulated in Table 1.

**Table 1:** Simulated cut-off points of the *DMCEs* statistic (*α* = 1.5, *β* = 1.5,*ω* = 0.5).

*n*	Level of percentiles	*κ* = 5	*κ* = 10	*κ* = 20
10	10%	0.0855	0.0697	0.0589
	5%	0.0940	0.0818	0.0716
	1%	0.1000	0.0985	0.0964
20	10%	0.0400	0.0298	0.0170
	5%	0.0457	0.0376	0.0283
	1%	0.0500	0.0479	0.0428
30	10%	0.0245	0.0162	0.0109
	5%	0.0281	0.0195	0.0118
	1%	0.0330	0.0295	0.0212
50	10%	0.0142	0.0098	0.0068
	5%	0.0154	0.0105	0.0073
	1%	0.0193	0.0131	0.0084
70	10%	0.0102	0.0072	0.0050
	5%	0.0113	0.0076	0.0054
	1%	0.0136	0.0089	0.0060
100	10%	0.0074	0.0051	0.0036
	5%	0.0079	0.0055	0.0038
	1%	0.0090	0.0059	0.0043
150	10%	0.0051	0.0036	0.0025
	5%	0.0054	0.0038	0.0027
	1%	0.0062	0.0042	0.0029

In general, for these particular choices of parameter values, the value of cut-off point decreases as the concentration parameter *κ* increases for all *n* and percentile levels. Similarly, as the sample size increases, the cut-off points decrease for all percentile levels and concentration parameters. The cut-off points may differ for different combinations of parameter values and are available upon request from the authors. Alternatively, the relevant program to obtain the cut-off points can be found at http://cran.r-project.org/.

### Power of Performance of the *DMCEs* Statistic

It is of interest to investigate the performance of the *DMCEs* statistic via simulation study. A similar scheme used in Section 4 is employed here. We introduce an outlier in the simulated data at point *d* of the response variable *v*, *v*_*d*_, such that

$$v_{d}^{*}=v_{d}+\lambda \pi \text{ mod}\left(2\pi \right)$$

where $v_{d}^{*}$ is the contaminated observation at position *d* and *λ* is the degree of contamination, 0 ≤ λ ≤ 1.

When *λ* = 0, there is no contamination at position *d*, whereas when *λ* = 1, the observation $v_{d}^{*}$ is located at the anti mode of its initial location. The generated data are fitted using Eq (2) and consequently we obtain the fitted values $\hat{v}$. Then, we calculate the value of *DMCEs* for each simulated data set. The statistic has good power of performances if the fraction of correctly detecting outlier at position *d* is close to 1.

**Fig 1:**
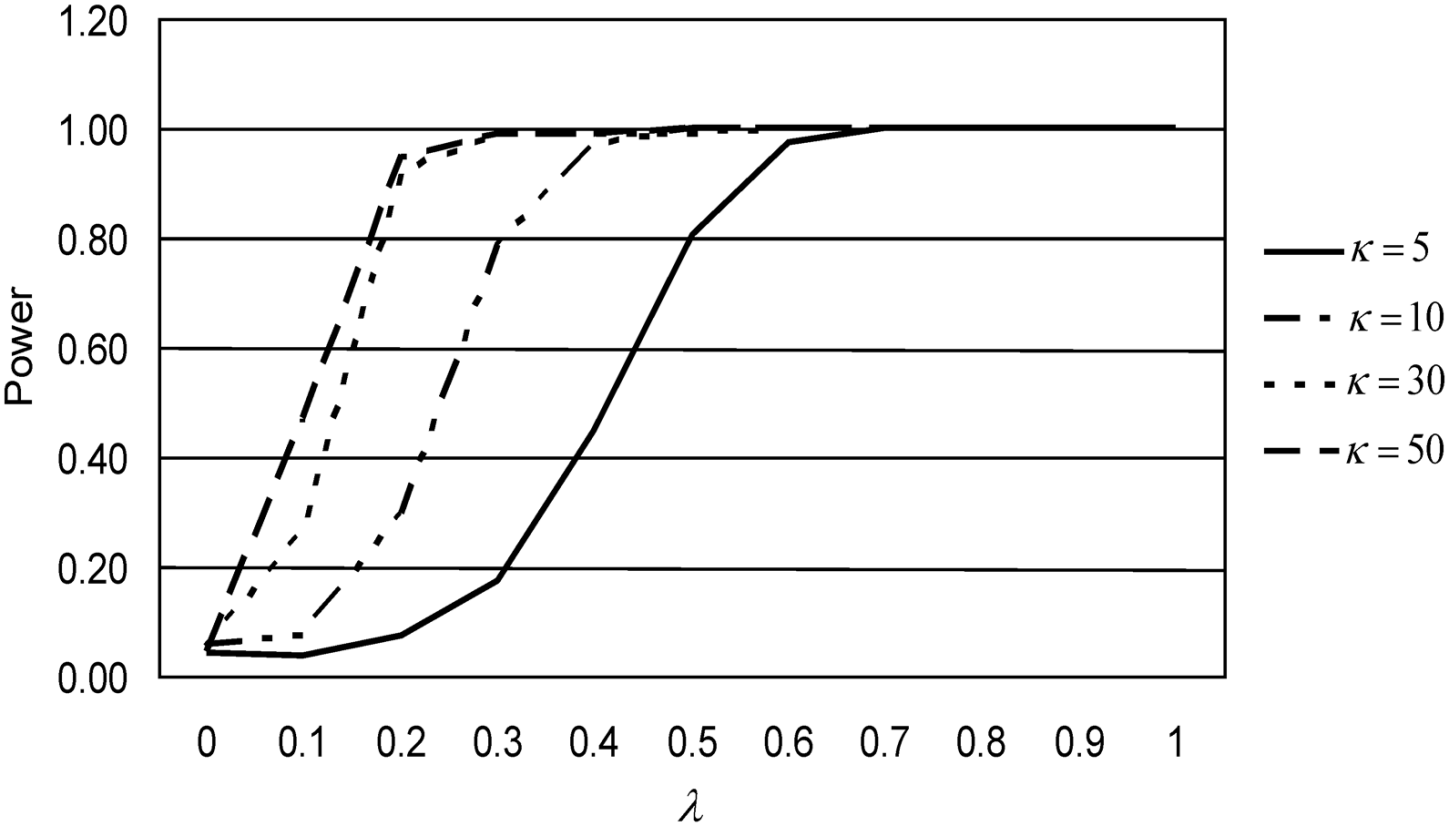
Power of performance of *DMCEs* statistic, for *n* = 70.

**Fig 2:**
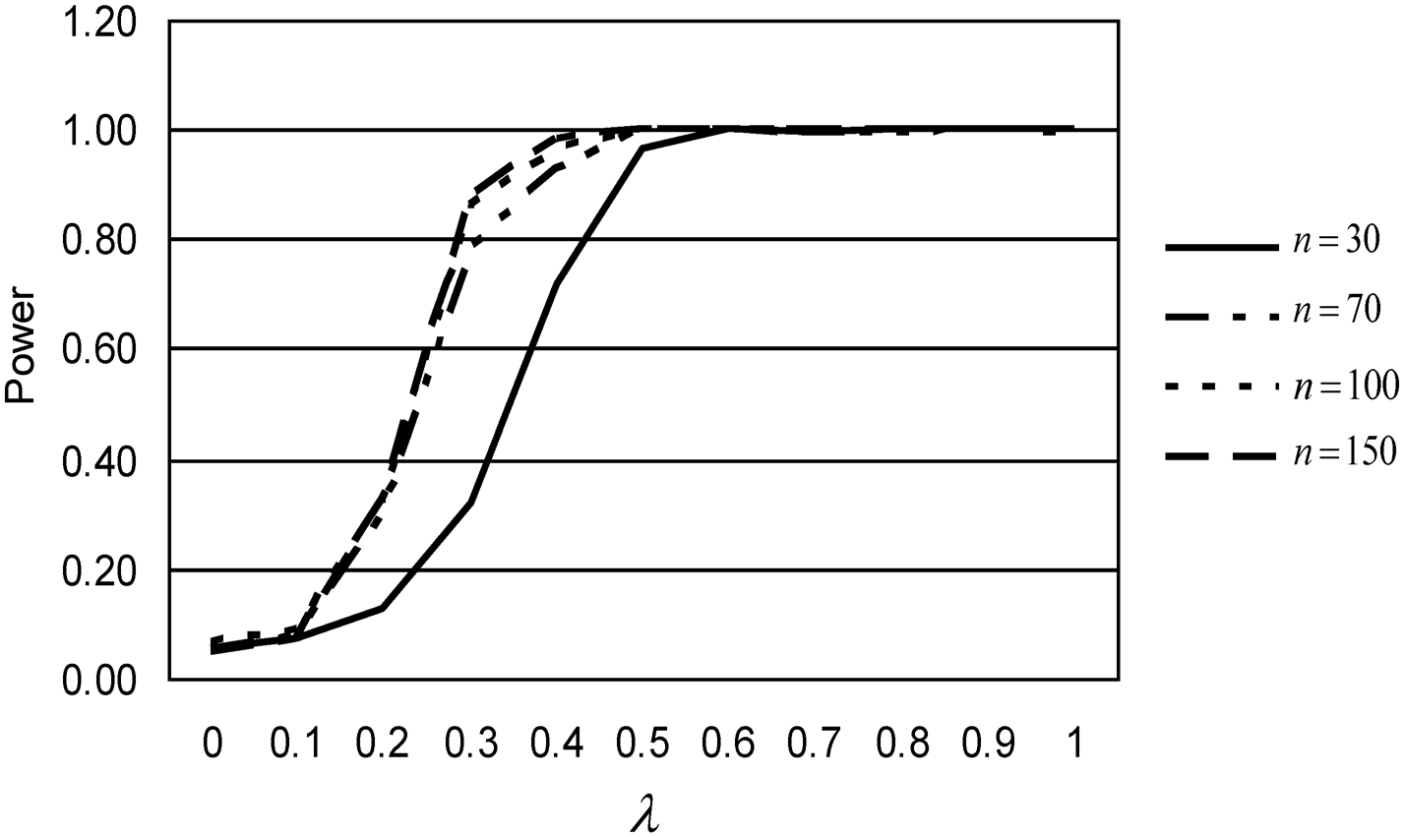
Power of performance of *DMCEs* statistic, for *κ* = 10.

Fig 1 shows the performance of *DMCEs* for *n* = 70 and various values of *κ*. When larger values are used, the performance is almost similar, but clearly better than that for small *κ*. On the other hand, Fig 2 gives the plot of the power of performance of the *DMCEs* statistic for *κ* = 10 and various sample sizes. We observe that the power of performance is an increasing function of sample size *n*. The *DMCEs* statistic performs better for larger sample size. Similar results are observed for the other cases.

## Real Example

### Background

Here we consider a real data set to show the estimation of the DM circular regression model using MLE method and the application of the *DMCEs* statistic using circadian data provided by Downs and Mardia [11]. The data are obtained from 10 medical students in Austria. The students are measured several times daily for a period of several weeks. The study period was split into two prime time periods as part of the study, and the peak time for systolic blood pressure (in degree) was estimated separately for each student for each period, giving values S1 and S2. The two blood pressure peak times should be equivalent, if circumstances are the same for each of the two periods.

### Descriptive Statistics

**Fig 3:**
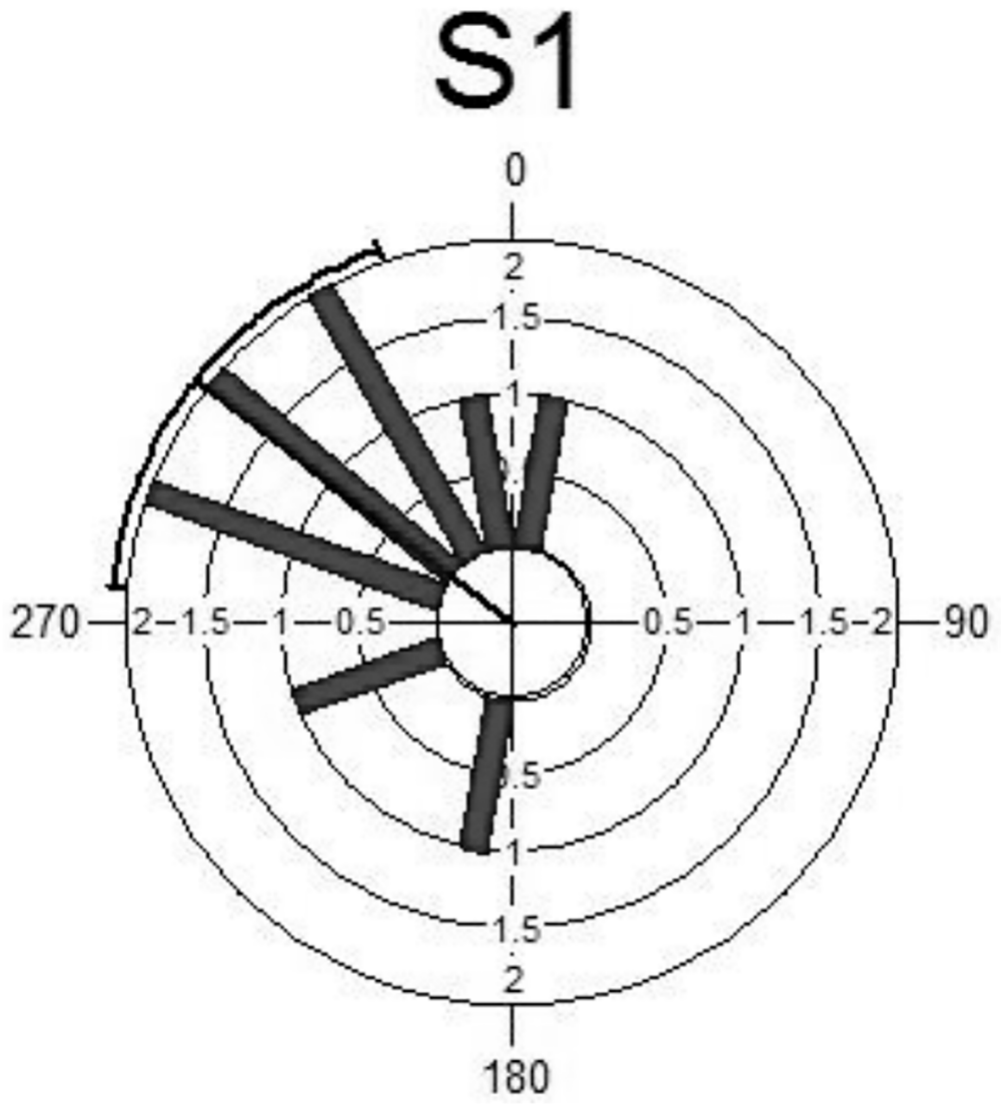
Circular Histogram for S1.

**Fig 4:**
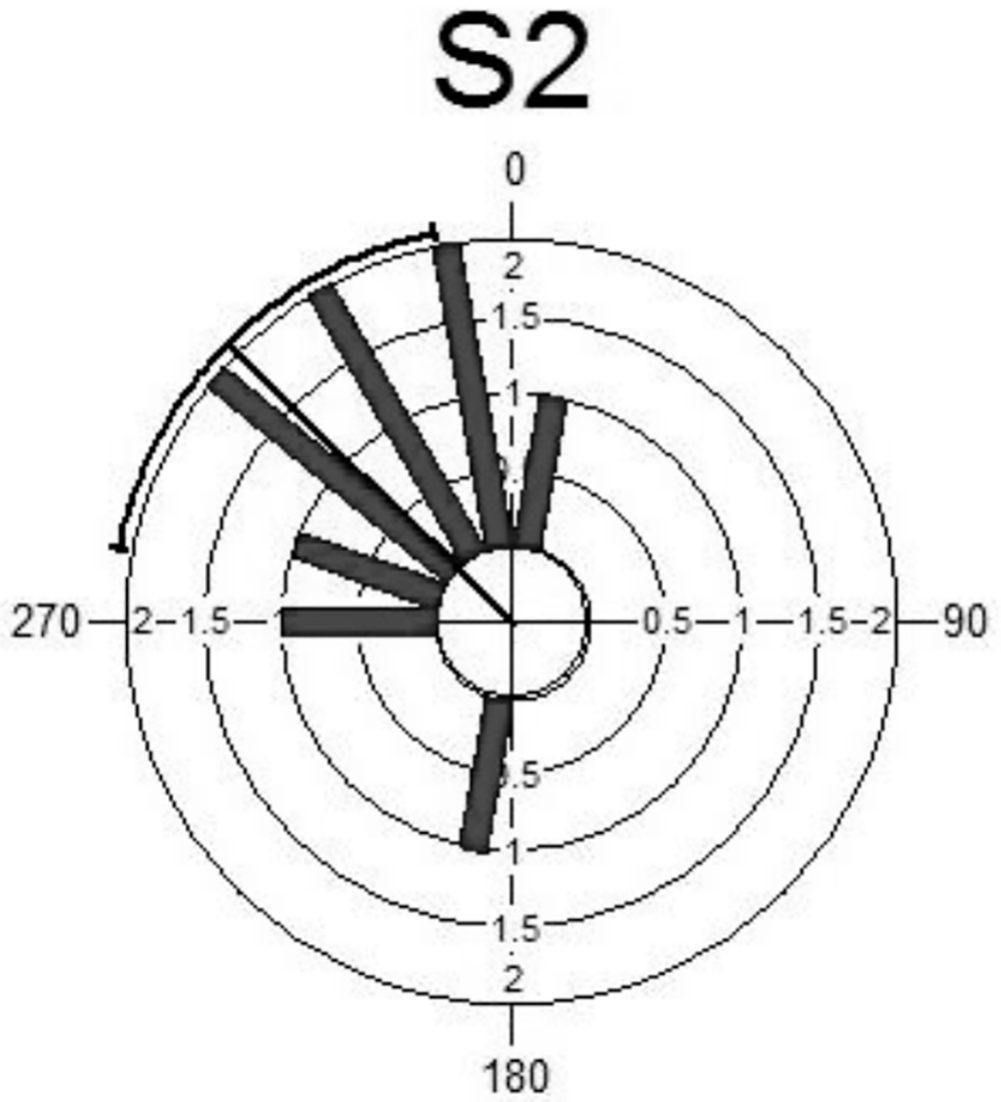
Circular Histogram for S2.

Several plots can be used to illustrate the distributions of both measurements. In general, from Figs 3 and 4, both sets of measurement follow the same distribution. It can be seen that the maximum blood pressures are observed in the upper left quadrant of the circular histogram indicating the same time in both periods. Some of the descriptive statistics for the circadian data are given in Table 2. The summary statistics of the S1 and S2 are almost similar including the concentration parameter with the value more than two.

**Table 2:** Descriptive statistics for circadian data.

Variable	S1(*u*)	S2(*v*)
Mean Direction	307.93°	314.69°
Mean Resultant Length	0.74	0.72
Circular Std Dev	44.87°	46.6°
Median Direction	314.5°	318°
Concentration parameter	2.251	2.125

**Fig 5:**
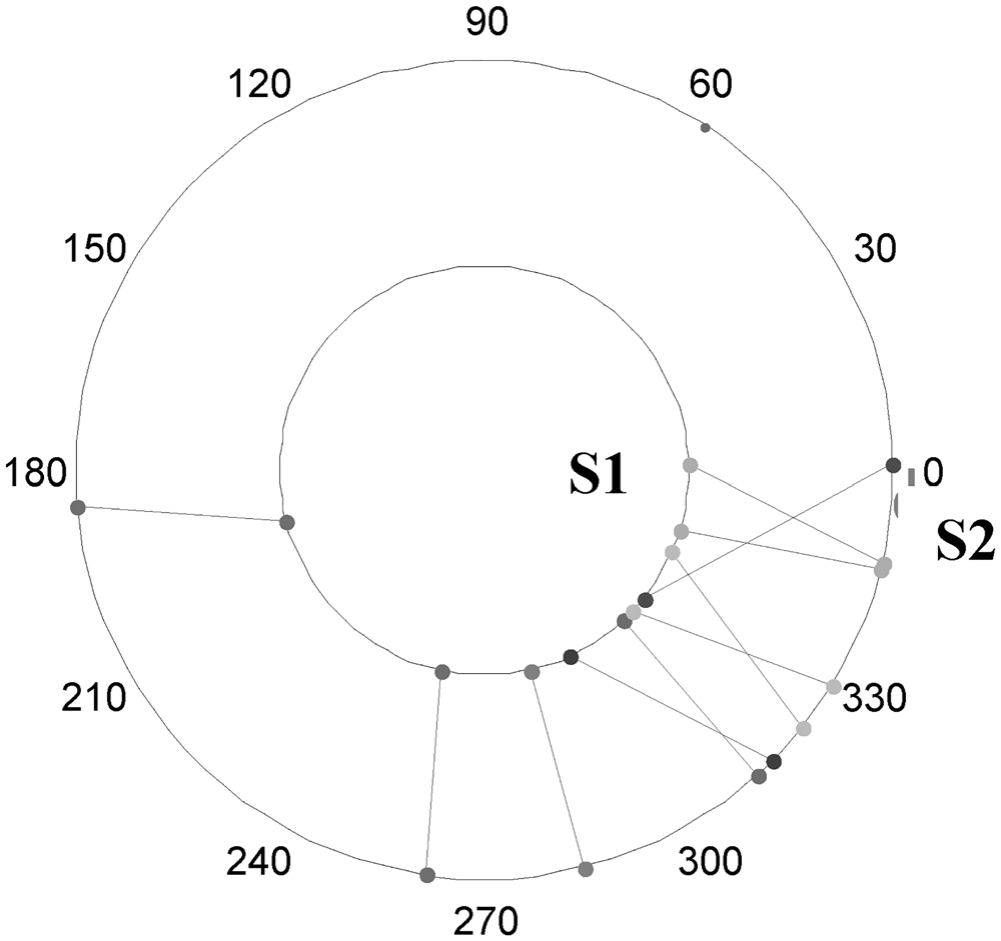
Spoke plot of circadian data.

In addition, Fig 5 shows the spoke plot of the data. By taking the horizontal axis in the right direction as 0°, the inner ring places the observations of S1 while the outer ring for S2. The lines connecting points on outer and inner rings correspond to the observed values of S1 and S2 respectively for the same time point. It can be observed that one line corresponding to student number 8 on the left hand side of the plot lies a distance away from the others.

### Parameter Estimation

**Fig 6:**
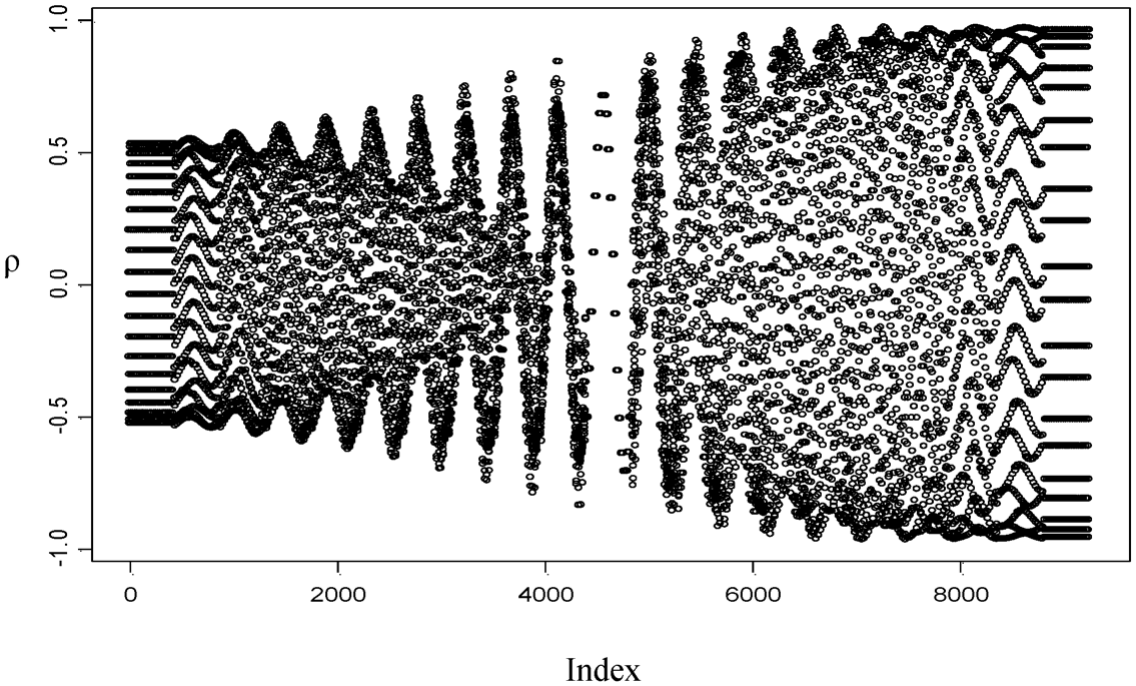
Plot of *ρ* versus index for circadian data.

Using the circadian data set, we calculate the precision parameters in the pre-specified sets as described in Section 2. The resulting plot of *ρ* versus index is given in Fig 6. The initial values of each parameter correspond to the highest point observed in the plot giving *α*_*o*_ = 18°, *β*_*o*_ = 9° and *ω*_*o*_ = 0.70. Thus, using these initial values, the final parameter estimates are obtained by maximizing the log likelihood function given by Eq (5): $\hat{\alpha }=16.58{}^{\circ}$, $\hat{\beta }=5.74{}^{\circ}$ and $\hat{\omega }=0.67$.

### Outlier detection

We now apply the outlier detection procedure based on the *DMCEs* statistic on the data. The student number 8 is flagged as a candidate of outlier. By employing the *DMCEs* statistic which uses the row deletion approach, such outlier is also known as an influential observation. The data is of size *n* = 10 with the maximum likelihood estimate of concentration parameter $\hat{\kappa }=17.64$ giving the cut-off point to be used is 0.07. Upon calculating the *DMCEs* for the data, we have *DMCEs* = 0.09 which is greater than the cut-off point and conclude that student number 8 is an influential observation.

Further, we investigate the effect of this observation on the parameter estimates. After removing student number 8 from the data set, we notice that the values of $\hat{\alpha }$ and $\hat{\beta }$ increase by a large value in degree while $\hat{\omega }$ also changes from 0.669 to 0.820 as shown in Table 3. Further investigation should then be carried out as the identification of this outlier might lead to useful understanding of the data.

**Table 3:** Effect of influential observation on parameter estimates.

Data	$\hat{\alpha }$	$\hat{\beta }$	$\hat{\omega }$
With the 8^th^ observation	16.57°	5.74°	0.67
Without the 8^th^ observation	51.02°	39.98°	0.82

## Conclusions

In this paper, we consider the problem of detecting outliers in the Down and Mardia’s circular regression model based on the *DMCEs* statistic. The sampling behaviour and the performance of the procedure are investigated via simulation. We illustrate the use of the new procedure using the circadian data set. In the future, it is our interest to introduce a more robust approach in identifying outliers by extending methods used in the linear case to circular.

## References

[1] Barnett V and Lewis T (1984), “Outliers in statistical data,” John Wiley & Sons, New York.

[2] Belsley DA, Kuh E, and Welsch RE (1980) “Regression Diagnostic: Identifying influential data and sources of collinearity,” John Wiley & Sons, New York; Chichester.

[3] Laycock PJ 1975 “Optimal regression: regression models for directions,” Biometrika, 62: 305–311.

[4] Gould AL (1969) “A regression technique for angular response,” Biometrics, 25: 683–700. 5362284

[5] Fisher NI and Lee AJ (1992) “Regression models for an angular response,” Biometrics, 48: 665–677.

[6] Johnson RA and Wehrly TE (1978) “Some angular-linear distributions and related regression models,” Journal of the American Statistical Association, 73(363): 602–606.

[7] Laycock PJ 1975 “Optimal regression: regression models for directions,” Biometrika, 62: 305–311.

[8] Rivest LP 1997 “A decentred predictor for circular –circular regression,” Biometrika, 84(3): 717–726.

[9] Jammalamadaka SR and Sarma YR (1993) “Circular Regression,” Statistical Sciences and Data Analysis, 109–128.

[10] Hussin AG, Fieller NRJ, and Stillman EC (2004) “Linear regression for circular variables with application to directional data,” Journal of Applied Science and Technology, 8: 1–6.

[11] Downs TD and Mardia KV (2002) “Circular regression,” Biometrika, 89(3): 683–697.

[12] Abuzaid AH, Mohamed IB, Hussin AG and Rambli A (2011) “COVRATIO statistic for simple circular regression model,” Chiang Mai J. Sci., 38(3): 321–330.

[13] Ibrahim S, Rambli A, Hussin AG, and Mohamed I. (2013) “Outlier detection in a circular regression model using COVRATIO statistic,” Communications in Statistics—Simulation and Computation, 42(10): 2272–2280.

[14] Abuzaid AH, Hussin AG and Mohamed IB (2013) “Detection of outliers in simple regression model using mean circular error statistic,” Journal of Statistical Computation and Simulation, 83(2): 269–277.

[15] Jammalamadaka SR and SenGupta A (2001) “Topics in Circular Statistics,” World Scientific, London.

